# Evidence for functional coupling of cGMP/cGKI signalling and TRPC channels in endothelium but not in vascular smooth muscle

**DOI:** 10.1186/2050-6511-14-S1-P40

**Published:** 2013-08-29

**Authors:** Florian Loga, Katrin Domes, Marc Freichel, Veit Flockerzi, Alexander Dietrich, Lutz Birnbaumer, Franz Hofmann, Jörg W Wegener

**Affiliations:** 1 FOR 923, Institut für Pharmakologie und Toxikologie, Technische Universität München, Germany; 2 Pharmakologisches Institut, Ruprecht-Karls-Universität, 69120 Heidelberg, Germany; 3 Experimentelle und Klinische Pharmakologie und Toxikologie, Universität des Saarlandes, Germany; 4 Institut für Pharmakologie und Toxikologie , Ludwig-Maximilians-Universität München, Germany; 5 National Institute of Environmental Health Sciences, North Carolina 27709, USA

## Background

Signaling via cGMP-dependent protein kinase I (cGKI) is the major pathway in vascular smooth muscle (SM), by which endothelial NO regulates vascular tone. Recent evidence suggests that canonical transient receptor potential (TRPC) channels are targets of cGKI in SM and mediate the relaxant effects of cGMP signaling. We tested this concept by investigating the role of cGMP/cGKI signaling on vascular tone and peripheral resistance using *Trpc*6^-/-^, *Trpc*3^-/-^, *Trpc*3^-/-^/*6*^-/-^, *Trpc*1^-/-^/3^-/-^/6^-/-^, and SM-specific cGKI^-/-^ (sm-cGKI^-/-^) mice.

## Results

α-adrenergic stimulation induced similar contractions in L-NAME-treated aorta and comparably increased peripheral pressure in hind limbs from all mouse lines investigated. After α-adrenergic stimulation, 8-Br-cGMP diminished similarly aortic tone and peripheral pressure in control, *Trpc*6^-/-^, *Trpc*3^-/-^, *Trpc*3^-/-^/6^-/-^, and *Trpc*1^-/-^/3^-/-^/6^-/-^ mice but not in sm-cGKI^-/-^ mice. In untreated aorta, α-adrenergic stimulation induced larger contractions in aorta from sm-cGKI^-/-^, *Trpc*6^-/-^, *Trpc*3^-/-^/6^-/-^, and *Trpc*1^-/-^/3^-/-^/6^-/-^ than in those from control and *Trpc*3^-/-^ mice indicating a functional link between cGKI and TRPC6 channels. TRPC3 channels were detected by immunocytochemistry in both isolated aortic SM cells (SMC) and aortic endothelial cells (EC), whereas TRPC6 channels were detected only in EC. Phenylephrine-stimulated Ca^2+^ levels were similar in SMC from Ctr and *Trpc*6*^-/-^* mice. Carbachol-stimulated Ca^2+^ levels were reduced in EC from *Trpc*6*^-/-^* mice. Stimulated Ca^2+^ levels were lowered by 8-Br-cGMP in Ctr but not in *Trpc6^-/-^* EC.

## Conclusion

The results suggest that cGKI and TRPC1,3,6 channels are not functionally coupled in vascular SM. Deletion of TRPC6 channels impaired endothelial cGKI signaling and the vasodilator tone in aorta.

